# Assessing the Management and Evaluation of Impacted Wisdom Teeth in a Dental Teaching Hospital

**DOI:** 10.3390/dj13020069

**Published:** 2025-02-03

**Authors:** Ahmed Al Subaie, Raghad Alruwili, Bayan Alkhuadher, Sarah Alzawwad, Wareef Alzamil, Khalifa S. Al-Khalifa

**Affiliations:** 1 Department of Biomedical Dental Sciences, College of Dentistry, Imam Abdulrahman Bin Faisal University, Damman 31441, Saudi Arabia; aialsubaie@iau.edu.sa; 2 College of Dentistry, Imam Abdulrahman Bin Faisal University, Damman 31441, Saudi Arabia; 2180003010@iau.edu.sa (R.A.); 2180005533@iau.edu.sa (B.A.); 2180004228@iau.edu.sa (S.A.); 2180003219@iau.edu.sa (W.A.); 3 Department of Preventive Dental Sciences, College of Dentistry, Imam Abdulrahman Bin Faisal University, Damman 31441, Saudi Arabia

**Keywords:** Saudi Arabia, impaction, third molar, evaluation, management

## Abstract

**Background**: Untreated impacted wisdom teeth can lead to complications, including delayed healing and inferior alveolar nerve damage. Delayed management is further complicated by age-related comorbidities. **Aim**: This study aimed to evaluate the management of impacted wisdom teeth in a teaching dental hospital and identify challenges faced by dental care providers. **Materials and Methods**: A retrospective radiographic study was conducted using data from electronic medical records and panoramic radiographs of patients. Independent variables included demographics and clinical details of wisdom teeth, such as type and presence of impaction, associated pathology, nerve proximity, second molar resorption, and extraction difficulty. The study assessed the evaluation of dental care provider practices in managing impacted wisdom teeth. Chi-square tests analyzed relationships between management type, provider level, and extraction difficulty. **Results**: Data from 270 panoramic radiographs and dental records were analyzed. Most cases were not managed (*n* = 216, 80%), irrespective of extraction difficulty or care provider level (undergraduate vs. graduate). There was no significant association between care provider levels and the type of management or between impaction difficulty and management type (*p* > 0.05). **Conclusions**: The findings highlight a lack of proper evaluation and management of impacted wisdom teeth, regardless of the provider’s experience. In hospital settings, all care providers should ensure the timely evaluation and management of impacted wisdom teeth to prevent complications.

## 1. Introduction

Impacted third molars that are left untreated require annual follow-up that include orthopantogram to detect any odontogenic cyst/tumor development early. The National Health Services in the United Kingdom and Finland, as well as the American Public Health Association, no longer recommend routine elective extraction of wisdom teeth. In other words, surgical removal of impacted third molars should only be performed when it is associated with symptoms, pathology, or infection, especially when the patient is older than 25 years [1,2,3,4]. The American Association of Oral and Maxillofacial Surgery shares similar opinions on the management of wisdom teeth. According to their guidelines, symptomatic wisdom teeth or wisdom teeth that are associated with pathology should be extracted. On the other hand, asymptomatic and pathology-free wisdom teeth should be assessed annually, both clinically and radiographically, by knowledgeable and competent professionals in the management of impacted third molars [5].

There have been several studies conducted worldwide and locally on the prevalence of impacted third molars. The global prevalence of third molar impaction was found to be 24.4% [6]. In Saudi Arabia, a cross-sectional study was conducted in Jazan region. Out of 1200 radiographs, 291 patients (24.3%) had impacted third molars [7]. In a digital panoramic study that was conducted in the Eastern Province, it was found that out of the 1068 patients, 206 (19.3%) had at least one impacted third molar [8]. A review of 359 panoramic radiographs used in a retrospective study in the Western Province showed that there was pathology associated with 18.2% of impacted maxillary third molars and 31.5% of impacted mandibular third molars [9]. In a cross-sectional retrospective study conducted in Jeddah, 4000 panoramic radiographs and dental charts were evaluated. According to the study, the prevalence of impacted third molars was 19.2% [10]. A cross-sectional study was conducted to assess the prevalence of impacted third molars in the population of the Riyadh region. Data from 1695 panoramic radiographs of patients were used for the study. At least 240 out of the 1695 panoramic radiographs examined showed the presence of one or more impacted third molars [11]. These studies shed light on the prevalence of impacted third molars. However, no study specific to Saudi Arabia or internationally has been found in the literature that assesses the incidence of failure to properly evaluate an impacted third molar.

It is not uncommon for dentists or dental students to overlook the proper evaluation and management of impacted wisdom teeth, which can result in serious complications [12,13]. Asymptomatic wisdom teeth do not necessarily indicate the absence of pathology [14]. Therefore, a comprehensive oral examination should include a careful clinical assessment of the wisdom teeth’s location, angulation, periodontal health, the quality of the surrounding soft tissue, and the ability to maintain proper hygiene around them. Once the impaction of the wisdom teeth is confirmed, the findings should be communicated and documented properly with the patient and referred to oral surgery for management [5,15].

Delayed management of impacted wisdom teeth could increase the likelihood of potential complications, such as delayed healing and inferior alveolar nerve damage [11,16]. Moreover, older people are more likely to have comorbidities such as diabetes mellitus or cardiovascular diseases, which could potentially require medical consultation, and increase the likelihood of medical emergencies or infection [17]. Therefore, it is advised to extract impacted wisdom teeth as early as possible, ideally before 24 years of age [11].

There seems to be a lack of literature on current clinical practices by dental care providers regarding the evaluation and management of impacted third molars. The primary objective of this study was to evaluate the management and assessment of impacted wisdom teeth in a dental teaching hospital.

## 2. Materials and Methods

### 2.1. Study Design and Setting

This retrospective study was conducted at a dental hospital located in the Eastern Province of Saudi Arabia. The data were extracted from the electronic medical record and panoramic radiographs of patients who came to the dental clinic for treatment between September 2020 and May 2023. Ethical approval was obtained from the scientific committee of Imam Abdulrahman Bin Faisal University.

### 2.2. Study Participants

Patients were selected according to the following inclusion criteria: (i) patients who have at least one impacted wisdom tooth, (ii) presence of diagnostic panoramic radiographs. Patients under the age of 15 years, patients on chemotherapy and/or radiotherapy, and patients with osteoporosis, bleeding disorders, and immune diseases were excluded from the study.

### 2.3. Sampling and Sample Size

Based on a sample size calculation with the following criteria, a 5% margin of error, a 0.05 alpha level, and a 20% expected third molar impaction prevalence according to a study that was conducted in the Eastern province [8]. An estimated 246 cases were needed for this study. The sample was increased by 10% (around 270 cases) for statistical purposes.

### 2.4. Data Collection Procedure

The patients’ digital panoramic radiographs were examined in a standardized manner under good lighting conditions and using a standardized screen brightness and resolution to determine the frequency and position of impacted third molars with a MiPACS Dental Enterprise Viewer (LEAD Technologies Inc., Charlotte, NC, USA). Two examiners were calibrated with a gold standard (An experienced OMFS consultant). Line and measurement tools of the software for assessing panoramic radiographs were used to find the number of impacted third molars and their angulation. Inter-examiner and intra-examiner reliability were checked using statistical kappa analysis. The results were 84% for inter-examiner and 92% for intra-examiner agreement. The angle between the long axis of the third molar with respect to the long axis of the adjacent second molar using Winters classification [18,19].

### 2.5. Data Collection Tool

A pre-prepared data extraction sheet was used to extract the following independent variables information from the panoramic radiographs: 1— Presence of impaction; 2—Impaction type according to Winter’s classification (Vertical impaction: Angulation between −10° and 10°; Mesioangular impaction: Angulation between 11° and 79°; Horizontal impaction: Angulation between 80° and 100°; and Distoangular impaction: Angulation between −79° and −11°); 3—Presence of pathology; 4—Proximity to the nerve, 5—Resorption of the second molar and difficulty of extraction which are ((i) easy for vertical impaction; (ii) moderate for mesioangular impaction and presence of pathology; and (iii) difficult for horizontal and distoangular impaction and proximity to the nerve). Moreover, another pre-prepared data extraction sheet was used to extract the following independent variables information from the patient’s charts: 1—Patient age, 2—Gender, 3—Chief complaint, 4—Past medical history, 5—Level of care provider, 6—Clinical examination ((i) clinical type of impaction complete or not; (ii) pocket depth distal to the second molar; (iii) quality of soft tissue distal to the second molar; (iv) available space distal to the second molar; (v) carious or not; (vi) accessibility for cleaning). The dependent variable (outcome) was the evaluation and management of impacted wisdom teeth by dental care providers that include no management, extraction, recommendation, or referral. “Recommendation” refers to the advice or suggestion provided by dental care providers for further action regarding the management of impacted wisdom teeth. This could include advising the patient to monitor the condition, consider conservative management (such as pain relief), or seek a second opinion or specialist consultation.

### 2.6. Statistical Analysis

Statistical analysis was performed using the Statistical Package for the Social Sciences Software (SPSS) Version 22. Descriptive statistics were in the form of frequencies and percentages. Bivariate analysis was performed using chi-square test to assess the relationship between the dependent variable (management) with the other independent variables. *p* < 0.05 was considered statistically significant.

## 3. Results

In the study sample, the percentage of male patients with impacted third molar was slightly higher than that of the female patients (54.4%). However, the majority of impaction (53.7%) was in the age range of 16–25 years. Most of the patients (88.1%) were medically fit and their chief complaint was not related to third molar (87.4%). Impacted third molar with no pathology constituted about 71.5% of the sample, 65.6% of them were close to the nerve, and only one third (33.7%) caused resorption of the second molar. Interestingly, 222 out of 270 (82.25%) were not evaluated according to the criteria of the clinical examination (Table 1). The majority of upper third molar impactions was vertical (27%), while it was mesioangular for the lower third molars (34.4%) (Table 2). The results of the independent variables demonstrate that 82.2% of the study sample size had no comprehensive clinical examination performed for impacted wisdom teeth.

There were no differences between care provider levels (undergraduate vs. postgraduate) in managing third molar impaction, as seen in (Table 3). Most of the cases (80%) were not managed, regardless of the difficulty of extraction and care provider level (Table 4). There was no association observed between care provider level and type of management, nor between the difficulty of impaction and type of management, using the chi-square test (*p* > 0.05).

## 4. Discussion

The present study aimed to assess the evaluation and management of impacted wisdom teeth in a teaching dental hospital in the Eastern Province of Saudi Arabia. Patient records provide insights into the importance of proper evaluation and management of impacted third molars, the complications encountered because of delayed management, and the overall postoperative outcomes if wisdom teeth are properly managed in a timely manner. In a teaching dental hospital, impacted wisdom teeth represent a common clinical concern, as revealed by the findings of our study [8].

The high proportion of impacted third molars necessitates a need for careful evaluation and appropriate management strategies [20]. The prevalence of wisdom teeth impaction in KSA was found to be 25.35% [14], which implies that wisdom teeth are frequently encountered during dental practice. Thus, comprehensive clinical and radiographic wisdom teeth assessment must be conducted [21,22].

In this study, we found that more than 80% of impacted wisdom teeth were not comprehensively assessed, as indicated by radiographic evidence of third molar impaction, with no documented evidence of comprehensive wisdom teeth assessment, treatment options discussion, proper referral to specialist, or management. This could be explained by the fact that most of the dental students and practitioners focus on patients’ chief complaints and miss looking for the potentially impacted wisdom teeth [14]. Comprehensive examinations of wisdom teeth that include type of impaction, periodontal assessment of the adjacent teeth, space distal to the second molar, quality of soft tissue distal to the second molar, and accessibility to cleaning [13] were not documented in the most of sample included in the study. This could be explained by the fact that most of the impacted wisdom teeth in this study were asymptomatic (more than 80%) and pathology-free (more than 75%). This could also be explained by the fact that dental students’ clinical requirements do not include the evaluation or extraction of wisdom teeth. Moreover, wisdom teeth extraction is only performed by oral surgeons or oral surgery residents, and the total number of oral surgery residents and oral surgeons is below the benchmark. The previous oral surgery didactic curriculum aimed to teach dental students the full scope of oral and maxillofacial surgery, including procedures such as exodontia, wisdom teeth assessment and management, and facial trauma. However, the clinical curriculum primarily focused on teaching students how to perform simple exodontia, without providing opportunities for clinical recruitment or assessment of wisdom teeth management. This gap in clinical training has led to a lack of proficiency among students and practitioners in assessing and managing impacted wisdom teeth. Additionally, a shortage of oral surgery staff in the dental hospital has contributed to this issue. With approximately 1000 patients visiting the dental hospital daily, and around 10% requiring oral surgery, the current staff of only 5 oral surgeons is insufficient. Ideally, at least 12 oral surgeons would be needed to meet the demands. To address these challenges, the new curriculum requires each student to perform a comprehensive assessment and management of impacted wisdom teeth, ensuring better training and proficiency in this critical area of oral surgery. Another reason for the lack of management of impacted wisdom teeth could be patients’ dental anxiety related to undergoing surgical procedures. A previous study showed that dental anxiety was higher in patients with impacted wisdom teeth who required surgical extraction, compared to others with non-impacted teeth requiring conventional dental extraction [23].

If impacted wisdom teeth are present, a comprehensive examination must be performed. Otherwise, the patient must be referred to an oral surgeon for further assessment and management [24]. If patients’ assessment reveals any wisdom teeth indications for extraction, the patient should be referred to a specialist for the removal of their third molars. These indications include pain, pericoronitis, periodontal disease (i.e., deep pocket >5 mm, loss of attachment), caries, difficulty in cleaning, interference with prosthesis, orthodontic tooth movement, orthognathic surgeries, and association with cysts or tumors [25]. Although asymptomatic and pathology-free impacted third molars may not require any surgical intervention, comprehensive clinical evaluation, adequate documentation, and lifelong follow-up must be provided [14]. The rationale behind long-term follow-up is that delayed assessment and management of impacted wisdom teeth were shown to be associated with several complications, such as periodontal disease around the impacted and adjacent teeth, resorption of the adjacent teeth, development of odontogenic infections (i.e., Ludwigs Angina), cysts (i.e., odontogenic keratocyst), and tumors (ameloblastoma). And early detection of those complications would ease the management and minimize the morbidity [14].

Demographic information of the patients included in this study showed a mean age of 28.4 years, which is considered a young sample. This indicates that impacted wisdom teeth are frequently encountered during early adulthood or late adolescence. Early evaluation and management of the wisdom teeth (≤25 years) was shown to be associated with less morbidity, such post operative discomfort, trismus, swelling, and infection. On the other hand, delayed extraction of impacted wisdom teeth was shown to be associated with increased post-operative discomfort, pain, swelling, and infection [13]. Other studies indicate that once an individual becomes older, the wisdom teeth become more difficult to extract and associated with more bone defects distal to the second molar [26]. In fact, other studies support the early extraction of wisdom teeth during adolescence. Additionally, these extractions may offer long-term benefits, as performing them before complete root formation can help minimize complications and enhance clinical outcomes [27].

The impacted wisdom teeth identified in this study were classified according to Winter’s classification [14]. We chose to use Winter’s classification primarily due to its simplicity and widespread use in clinical practice, which allowed for easier comparison of cases. However, we acknowledge that the Pell and Gregory classification provides a more detailed evaluation of impaction depth and its relationship to surrounding structures. The decision not to include this system was based on the scope of the study and the focus on specific clinical outcomes rather than a comprehensive analysis of impaction depth. The majority of the pattern of impaction in our study was vertical for maxillary wisdom teeth, while it was mesioangular for lower wisdom teeth. These findings are similar to those in a study conducted in Riyadh [14]. The type of impaction contributes to the difficulty of extraction and, consequently, the overall outcome, with mesioangular impactions being associated with higher success rates compared to distoangular and vertical impactions [28]. These findings are consistent with previous studies that have shown that mesioangular impactions are easier to manage compared to other types of impactions [29].

It is worth mentioning that the majority (approx. 85%) of impacted wisdom teeth (based on retrospective radiographic assessment) in our study were considered difficult to extract. This may explain why most of the impacted wisdom teeth were not removed by students and non-oral surgery specialists. However, although the retrospective radiographic analysis identified a high proportion of difficult extractions, the lack of proper clinical evaluation in the medical records may preclude this factor from being used as a sole explanation for why many impacted wisdom teeth were not managed by students or non-oral surgery specialists. To assess the difficulty of wisdom teeth extraction, various factors need to be considered, such as impaction depth, angulation, and relationship to the ramus in the mandible [30]. Additionally, the Pederson index can be utilized to assess the difficulty of the surgery [31,32]. The depth of the impacted teeth significantly affects the complexity of extraction, with deeper impactions often presenting greater challenges [33]. Moreover, the difficulty of the surgery can be impacted by the relationship between impacted teeth and adjacent structures, such as the inferior alveolar nerve, which can lead to postoperative complications such neurosensory disturbance to the chin and lip [34]. Evaluating the difficulty of wisdom teeth extraction also involves considering the impact on surrounding structures. For example, the extraction of impacted mandibular third molars can influence the periodontal status of adjacent teeth, such as the second molar [34]. Proper assessment of the surgical difficulty and postoperative outcomes, including pain levels and stress, is crucial for successful wisdom teeth extraction [35,36]. In cases where extraction is challenging, techniques like bone grafting may be considered to assist in soft tissue wound healing and bone preservation [37]. Proper wisdom teeth assessment relies on key elements, such as obtaining focused history about previous wisdom teeth discomfort, pain, swelling, pus discharge, or infection. It also relies on comprehensive clinical evaluation of wisdom teeth and the surrounding area, proper radiograph acquisition and interpretation, and, consequently, reaching to accurate diagnosis. Once the third molar impaction is confirmed, the practitioner should be able segregate asymptomatic ones from symptomatic or pathology-associated ones. Symptomatic and pathology-associated wisdom teeth and wisdom teeth that interfere with orthodontics, orthognathic surgery, or prosthesis should be removed by the practitioner or referred to a specialist for further assessment and management. Asymptomatic wisdom teeth could either be managed conservatively with yearly follow-ups and panoramic radiographs or extracted based on a comprehensive discussion with the patient.

The data of this study revealed that almost 30% of impacted wisdom teeth caused resorption of the second molars. This could be due to the lack of accuracy of panoramic radiographs. This highlights the importance of thorough clinical and radiographic assessments and early detection strategies to prevent this complication. Thus, we recommend that patients showing questionable radiographic evidence of root resorption or any pathology are required to undergo an immediate CBCT scan to confirm or rule out the finding. If the resorption or pathology is confirmed, the patient will be informed of the result and referred to the oral surgery department for further management.

Our study has some limitations that should be considered when interpreting the results. First, panoramic radiographs were used as a preliminary tool for evaluation; however, there is a need for more advanced imaging techniques, such as CBCT, for more accurate and detailed assessments. Also, the study was conducted retrospectively in a single center, which may have introduced bias in the selection of cases and limited the generalizability of the findings. Another limitation could be the data collection methods utilized in this study, such as the reliance on medical records, which could introduce inaccuracies or missing information. Furthermore, the tools used to measure outcomes or variables in this study might have limitations in terms of accuracy, reliability, or sensitivity, which could affect the validity of the results. Future prospective studies with multi-center and longer follow-up periods would provide more robust evidence and facilitate the evaluation of treatment outcomes over time [38].

Despite some minor limitations, the findings of this study clearly emphasize the lack or under-reporting of comprehensive and systematic wisdom teeth evaluation. Accordingly, more comprehensive evaluation and management must be strictly implemented. Also, all radiographs must be interpreted comprehensively, irrespective of the reason for the radiograph or the patients’ chief complaints. If wisdom teeth impaction is encountered, it should be managed in a timely manner. Moreover, it is recommended that evaluation and management of impacted wisdom teeth be part of continuous education programs [39]. We also emphasize the need for integrating the evaluation and management of impacted wisdom teeth into dental school clinic curricula. This will help ensure that dental students are adequately trained to address these issues early on, potentially reducing the risk of complications and improving patient outcomes in the long term.

## 5. Conclusions

The findings reveal a significant gap in the evaluation and management of impacted wisdom teeth, which may be attributed to a shortage of oral surgery staff and a clinical curriculum that primarily focuses on simple exodontia. This underscores the critical need for adequate oral surgery staffing and the integration of comprehensive wisdom teeth assessment and management into the clinical training requirements. Delayed management of impacted wisdom teeth could increase the likelihood of potential complications. Therefore, it is advised to properly evaluate impacted wisdom teeth as early as possible, document the findings, communicate them with the patient, and manage accordingly.

## Figures and Tables

**Table 1:** Descriptive statistics of study variables.

Group	Variables	Values (*n* = 270)	N	%
Patient information	Gender	Male	147	54.4
		Female	123	45.6
	Age	16–25 years	145	53.7
		26–35 years	38	14.1
		Over 36 years	87	32.2
	Past medical history	Medically fit	238	88.1
		Medically compromised	32	11.9
	Chief complaint	Wisdom teeth related	34	12.6
		Not wisdom teeth related	236	87.4
	Presence of pathology	Present	77	28.5
		Not present	193	71.5
	Proximity to the nerve	Close	177	65.6
		Far	93	34.4
	Resorption of second molar	Present	91	33.7
		Not present	179	66.3
Care provider	Clinical examination	All the 6 criteria were achieved	2	0.7
		Some of the 6 criteria were achieved	46	17
		None of the 6 criteria were achieved	222	82.2
	Care provider level	Undergraduate	128	47.4
		Graduate	142	52.6
	Type of management	No management	216	80
		Extraction	22	8.1
		Recommendation	6	2.2
		Referral	26	9.6
	Difficulty of extraction	Easy	22	8.1
		Moderate	20	7.4
		Difficult	228	84.4

**Table 2:** Type of impaction of wisdom teeth.

Tooth #	Type of Impaction N (%)					
	Missing	Vertical	Mesioangular	Horizontal	Distoangular	Erupted
**Tooth #1**	61 (22.6%)	73 (27%)	24 (8.9%)	1 (0.4%)	52 (19.3%)	59 (21.9%)
**Tooth #16**	52 (19.3%)	66 (24.4%)	25 (9.3%)	4 (1.5%)	63 (23.3%)	60 (22.2%)
**Tooth #17**	43 (15.9%)	39 (14.4%)	93 (34.4%)	37 (13.7%)	5 (1.9%)	53 (19.6%)
**Tooth #32**	45 (16.7%)	43 (15.9%)	91 (33.7%)	34 (12.6%)	7 (2.6%)	50 (18.5)

**Table 3:** The relationship between care provider level and the type of management.

		Type of Management N (%)				Total
		No Management	Extraction	Recommendation	Referral	
**Care provider level**	**Undergrad**	104 (81.3%)	11 (8.6%)	3 (2.3%)	10 (7.8%)	128 (100%)
	**Grad**	112 (79.4%)	11 (7.8%)	3 (2.1%)	15 (10.6%)	142 (100%)
**Total**		216 (80.3%)	22 (8.2%)	6 (2.2%)	25 (9.3%)	270 (100%)

**Table 4:** The relationship between the difficulty of extraction and the type of management.

		Type of Management N (%)				Total
		No Management	Extraction	Recommendation	Referral	
**Difficulty of extraction**	**Easy**	18 (81.8%)	1 (4.5%)	1 (4.5%)	2 (9.1%)	22 (100%)
	**Moderate**	17 (85%)	1 (5%)	0 (0%)	2 (10%)	20 (100%)
	**Difficult**	181 (79.4%)	20 (8.8%)	5 (2.2%)	22 (9.6%)	228 (100%)
**Total**		216 (80%)	22 (8.1%)	6 (2.2%)	26 (9.6%)	270 (100%)

## Data Availability

The data are provided within the manuscript.
